# *Bacteroides fragilis* and *Microbacterium* as Microbial Signatures in Hashimoto’s Thyroiditis

**DOI:** 10.3390/ijms26178724

**Published:** 2025-09-07

**Authors:** Artur Kovenskiy, Nurlubek Katkenov, Aigul Ramazanova, Elizaveta Vinogradova, Zharkyn Jarmukhanov, Zhussipbek Mukhatayev, Almagul Kushugulova

**Affiliations:** 1 Laboratory of Microbiome, Center for Life Sciences, National Laboratory Astana, Nazarbayev University, 53 Kabanbay Batyr Ave., Block S1, Astana 010000, Kazakhstan; artur.kovenskiy@nu.edu.kz (A.K.);; 2 Department of Dermatovenereology and Dermatocosmetology, NJSC “Astana Medical University“, 50/2 Republic Ave., Astana 010000, Kazakhstan; 3 Interdisciplinary Sports Research, Center for Genetics and Life Sciences, Sirius University of Science and Technology, 1 Olympic Ave., Sirius Federal Territory, Sochi 354340, Russia

**Keywords:** microbiome, Hashimoto’s thyroiditis, alopecia areata, *Bacteroides fragilis*, autoimmunity

## Abstract

Hashimoto’s thyroiditis (HT) and alopecia areata (AA) are organ-specific autoimmune diseases that frequently co-occur, suggesting shared immunological and microbial pathways. The gut microbiome has emerged as a key modulator of immune function, yet disease-specific microbial signatures remain poorly defined. Fecal samples from 51 participants (HT: *n* = 16, AA: *n* = 17, healthy controls: *n* = 18) aged 18–65 years were analyzed using shotgun metagenomic sequencing followed by multivariate statistical analyses. While alpha and beta diversity did not differ significantly across groups, taxonomic profiling revealed disease-specific microbial patterns. *Bacteroides fragilis* was significantly enriched in HT, suggesting a potential role in immune modulation; although mechanisms such as polysaccharide A production and molecular mimicry have been proposed in previous studies, their involvement in HT remains to be confirmed. *Microbacterium* sp. *T32* was elevated in both HT and AA, indicating its potential as a shared autoimmune marker. Functional analysis showed increased fermentation and amino acid biosynthesis in AA, contrasting with reduced metabolic activity and elevated carbohydrate biosynthesis in HT. HT and AA exhibit distinct gut microbial and metabolic signatures. *Bacteroides fragilis* and *Microbacterium* sp. *T32* may serve as potential microbial correlates for autoimmune activity, offering new insights into disease pathogenesis and targets for microbiome-based interventions.

## 1. Introduction

Hashimoto’s thyroiditis (HT), the most common autoimmune thyroid disorder, is marked by lymphocytic infiltration of the thyroid gland, progressive destruction of thyroid follicles, and the presence of circulating autoantibodies, particularly anti-thyroperoxidase (anti-TPO) and anti-thyroglobulin (anti-TG) antibodies [1,2]. HT often manifests as hypothyroidism and is influenced by genetic, environmental, and microbial factors, including iodine intake and gut microbiota composition [3,4]. In addition to its systemic endocrine effects, HT often coexists with other autoimmune conditions, reflecting a broader immune dysregulation.

Among these associated disorders is alopecia areata (AA), a chronic autoimmune disorder characterized by non-scarring hair loss, which can range from discrete patches to complete scalp or body alopecia. While the precise pathogenesis remains unclear, AA is understood to involve autoreactive T cell-mediated damage to hair follicles, often triggered by stress, hormonal fluctuations, or genetic predisposition [5,6]. In addition to its significant psychological impact, AA frequently co-occurs with other autoimmune conditions such as vitiligo, psoriasis, and thyroid diseases, including HT [7].

The gut microbiome, comprising trillions of microorganisms residing in the gastrointestinal tract, plays a central role in regulating human metabolic, immune, and barrier functions. Dysbiosis, a disruption of the normal microbial balance, has been increasingly implicated in the onset and progression of autoimmune disorders [8,9,10]. The microbiome not only supports barrier integrity but also actively shapes the host immune system throughout life [10]. For example, segmented filamentous bacteria (SFB), a commensal group known to adhere to the intestinal epithelium, have been shown to drive IL-23/Th17-dependent mucosal and systemic immune responses, highlighting the influence of specific microbial taxa on autoimmune processes [11,12].

Emerging evidence suggests a shared immunological basis between HT and AA, two organ-specific autoimmune diseases that frequently co-occur. Genetic studies have demonstrated bidirectional associations between HT and AA, indicating that susceptibility to one condition may increase the risk of developing the other [13]. Both conditions involve dysregulation of the Th1/Th17–Treg axis and have been linked to gut microbial imbalances, pointing to the gut–immune interface as a possible contributor to disease pathogenesis [3,4,14]. Interestingly, thyroxine and melanin hormonal and pigmentary molecules affected in HT and AA, respectively, share a common biochemical precursor, tyrosine. This molecular overlap may play a role in the development of cross-reactive immune responses, suggesting a potential mechanistic link between HT and AA [15]. Understanding the microbial and immunological intersections between HT and AA may uncover common mechanistic pathways and guide the development of targeted microbiota-based interventions. This study aimed to compare gut microbiome diversity, identify disease-associated taxa, and explore functional pathways in Hashimoto’s thyroiditis and alopecia areata.

## 2. Results

Alpha and beta diversity analyses showed no significant differences in gut microbial diversity across the study groups (Supplementary Figure S1). Alpha diversity (Chao1 and Shannon indices) showed no significant differences among healthy controls (HC), alopecia areata (AA), and Hashimoto’s thyroiditis (HT), indicating comparable species richness and evenness across groups (Chao1 *p* = 0.16; Shannon *p* = 0.16). AA had the highest diversity, while HT showed a trend toward reduced richness and increased dominance by fewer taxa. Beta diversity (Bray–Curtis) and PCoA revealed overlapping microbial profiles (PC1 = 19.4%, PC2 = 9.2%), with no significant group separation (PERMANOVA F = 0.75, *p* = 0.911).

Comparative taxonomic profiling at the phylum level showed that microbial communities were predominantly composed of Firmicutes and Bacteroidota, which together accounted for the majority of relative abundance in all groups. At the genus level, the most abundant taxa included *Segatella*, *Faecalibacterium*, *Alistipes*, *Bacteroides*, and *Prevotella*. *Segatella* has trend toward elevation in HT group. On the species level, this trend is supported by *Segatella copri*.

**Figure 1:**
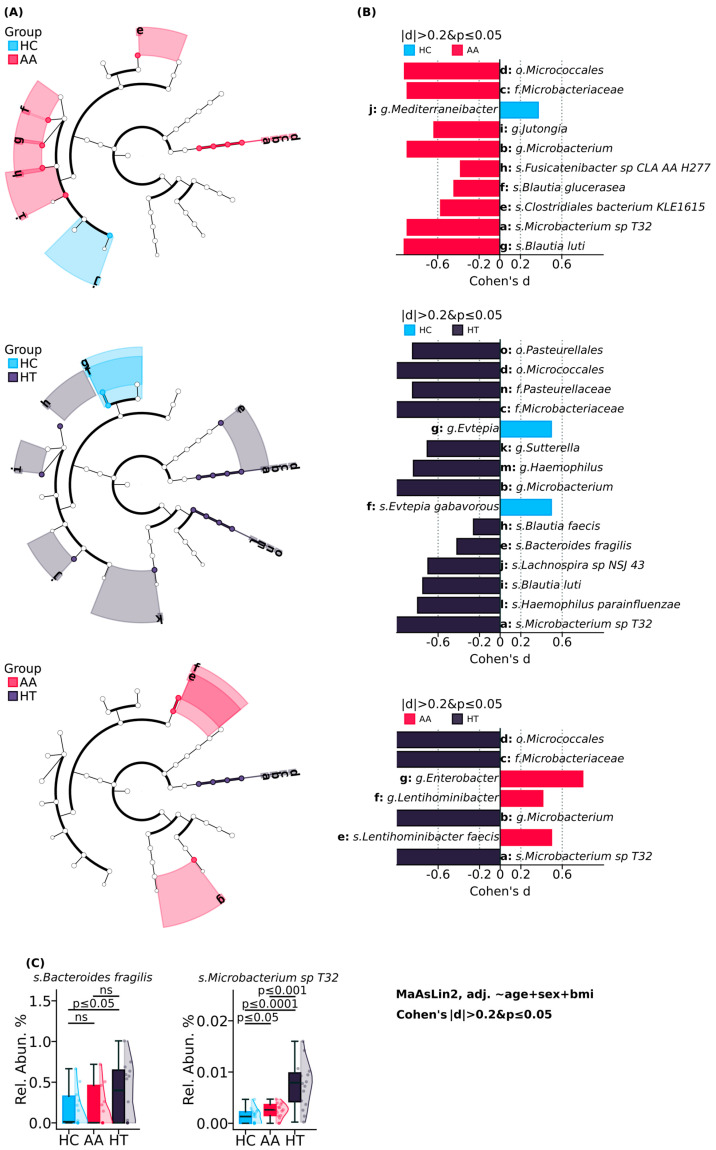
Gut microbiome compositional analysis comparing study cohorts. (**A**) The phylogenetic trees illustrating the hierarchical taxonomic difference between alopecia areata, Hashimoto’s thyroiditis, and healthy control groups. (**B**) Cohen’s d effect sizes of differentially abundant taxa between alopecia areata, Hashimoto’s thyroiditis, and healthy control groups. (**C**) Relative abundance of species *Bacteroides fragilis* and *Microbacterium* sp. *T32*.

Gut microbiome compositional analysis, illustrated in Figure 1, revealed distinct taxonomic signatures differentiating autoimmune conditions (AA and HT) from healthy controls, with *Microbacterium* sp. *T32* and *Bacteroides fragilis* identified as higher-abundance taxa alongside broader shifts across multiple phylogenetic levels.

Cladogram comparisons across AA, HT, and HC groups consistently identified significant enrichment of the Microbacteriaceae family, including *Microbacterium* sp. *T32*, *Microbacterium* (genus), and the order *Micrococcales* in both autoimmune groups relative to controls. MaAsLin2 differential abundance analysis confirmed these findings, highlighting *Microbacterium* sp. *T32* as a reproducibly elevated species in AA (*p* ≤ 0.05) and HT (*p* ≤ 0.0001) cohorts compared to HC.

In HT subjects, *Bacteroides fragilis* also showed a significant increase (*p* ≤ 0.05), further supported by MaAsLin plots indicating its consistent presence among key discriminatory taxa. Other notable shifts in HT included elevated *Haemophilus parainfluenzae*, *Lachnospira* sp. *NSJ_43*, and *Blautia faecis*. Meanwhile, the AA group exhibited enrichment in *Blautia glucerasea*, *Blautia luti*, *Fusicatenibacter* sp. *CLA_AA_H277*, *Jutongia*, and *Clostridiales bacterium KLE1615*.

In pairwise comparisons between AA and HT, genera such as *Lentihominibacter* and *Enterobacter* showed differential abundance, indicating AA-specific microbial trends within autoimmune disease spectra. While AA and HT shared several microbial alterations compared to HC, the prominence of *Microbacterium* sp. *T32* in both, alongside the selective elevation of *Bacteroides fragilis* and other opportunistic species in HT, suggests that overlapping yet distinct microbial signatures may underlie their respective immune pathophysiologies. These taxa may serve as candidates for further functional studies and potential microbial signatures correlated with autoimmune subtypes.

**Figure 2:**
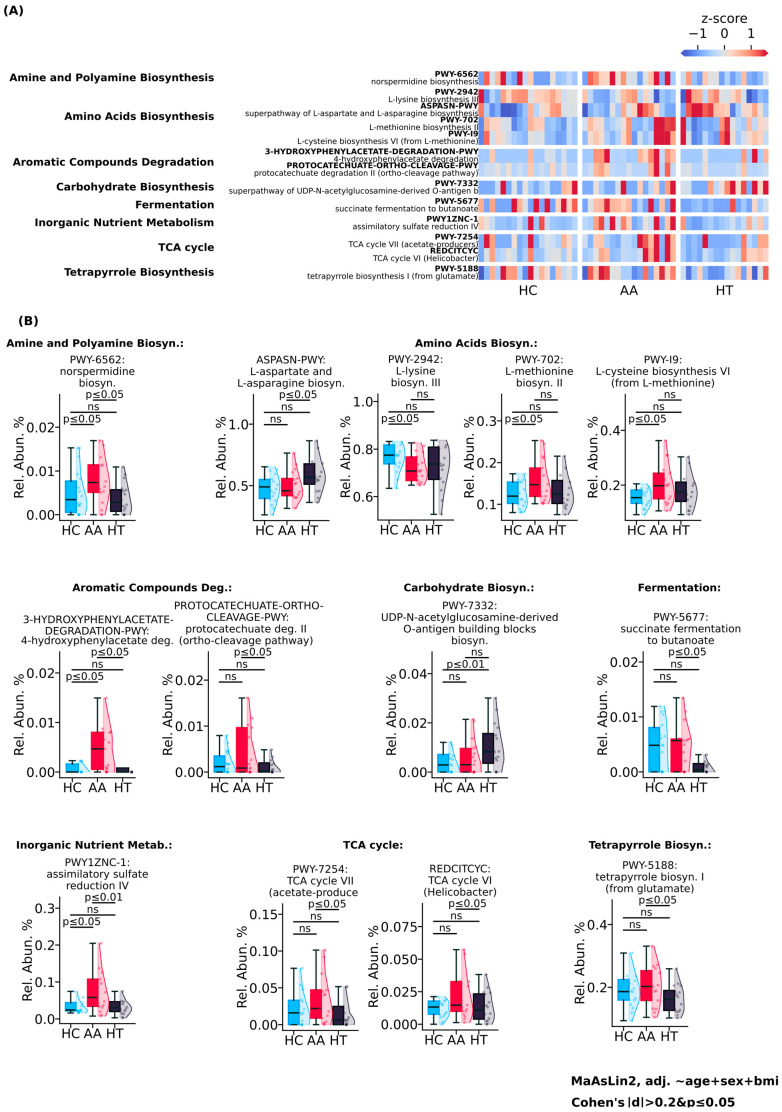
Functional analysis of microbial metabolic pathways comparing study groups. (**A**) Heatmap of metabolic pathway grouped by functional categories. (**B**) Box plots displaying significantly altered pathways.

Functional profiling of microbial metabolic pathways, showed in Figure 2, revealed a broadly elevated metabolic pattern in AA, contrasting with the reduced functional activity observed in HT. Degradation of aromatic compounds was markedly higher in AA and lower in HT, as evidenced by increased abundance of 4-hydroxyphenylacetate degradation and protocatechuate degradation II (ortho-cleavage pathway), suggesting enhanced microbial capacity for phenolic substrate breakdown in AA. Fermentation and inorganic nutrient metabolism were also more active in the AA group, with succinate fermentation to butanoate and assimilatory sulfate reduction IV pathways elevated 2-fold and 3-fold, respectively, compared to healthy controls.

TCA cycle activity differed between groups: TCA cycle VI (Helicobacter-type) was elevated in AA, while TCA cycle VII (acetate producers) was downregulated in HT. The tetrapyrrole biosynthesis I (from glutamate) pathway, involved in heme and cofactor production, was 1.25-fold more abundant in AA than in HT, further supporting increased biosynthetic output. Amino acid biosynthesis pathways also differed: L-methionine biosynthesis II and L-cysteine biosynthesis VI (from L-methionine) were enriched in AA, whereas HT was characterized by higher abundance of the superpathway of L-aspartate and L-asparagine biosynthesis. In contrast, L-lysine biosynthesis III was highest in healthy controls.

Interestingly, AA showed the lowest activity in amine and polyamine biosynthesis, with norspermidine biosynthesis reduced 3-fold compared to healthy controls. Meanwhile, HT exhibited a 3-fold increase in the superpathway of UDP-N-acetylglucosamine-derived O-antigen building blocks, suggesting enhanced microbial carbohydrate biosynthesis.

These findings demonstrate that AA is associated with a broadly activated gut microbial functional profile, including increased fermentation, aromatic compound degradation, and amino acid biosynthesis. In contrast, HT presents a more metabolically constrained signature, with selective upregulation of carbohydrate-related pathways, reflecting distinct patterns of microbial functional engagement across autoimmune diseases.

**Figure 3:**
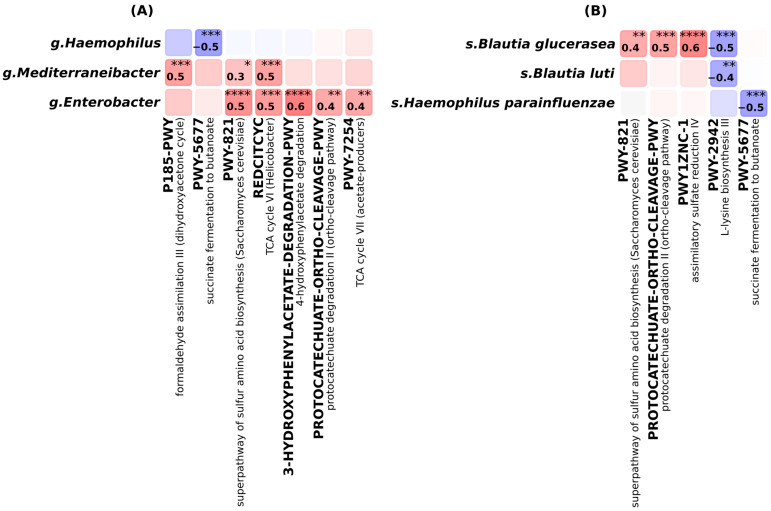
Spearman’s correlation between bacterial taxa and metabolic pathway. (**A**) At genus level. (**B**) At species level. * *p* ≤ 0.05, ** *p* ≤ 0.01, *** *p* ≤ 0.001, **** *p* ≤ 0.0001.

Correlation analysis, illustrated in Figure 3, between microbial taxa and functional pathways revealed coherent associations, highlighting taxon-specific contributions to metabolic activity within the gut microbiome of AA, HT, and HC groups. At the genus level, strong positive correlations (r ≥ 0.4) were observed between *Enterobacter* and several metabolic pathways. The most pronounced associations were found with aromatic compound degradation pathways, including 4-hydroxyphenylacetate degradation (r = 0.6) and protocatechuate degradation II (ortho-cleavage pathway) (r = 0.4). *Enterobacter* was also positively correlated with TCA cycle VII (acetate producers). Both *Enterobacter* and *Mediterraneibacter* showed positive correlations with the superpathway of sulfur amino acid biosynthesis (r = 0.5 and r = 0.3, respectively) and TCA cycle VI (Helicobacter-type) (r = 0.5, r = 0.5). Additionally, *Mediterraneibacter* was positively associated with formaldehyde assimilation III. In contrast, *Haemophilus* exhibited a strong negative correlation with succinate fermentation to butanoate (r = −0.5), indicating an inverse relationship with microbial fermentative activity.

At the species level, *Blautia glucerasea*, *Blautia luti*, and *Haemophilus parainfluenzae* emerged as functionally relevant taxa. Both *B. glucerasea* and *B. luti* were negatively correlated with L-lysine biosynthesis III (r = −0.5 and −0.4, respectively). In contrast, *B. glucerasea* showed strong positive correlations with the superpathway of sulfur amino acid biosynthesis (r = 0.4), protocatechuate degradation II (r = 0.5), and assimilatory sulfate reduction IV (r = 0.6), suggesting a multifunctional role in microbial biosynthesis and degradation. *H. parainfluenzae* was negatively correlated with succinate fermentation to butanoate (r = −0.5), reinforcing its association with reduced fermentative potential.

These taxon–function relationships are consistent with the pathway enrichment profiles observed in AA and HT and support the contribution of specific microbial taxa to condition-specific alterations in gut metabolic output.

**Figure 4:**
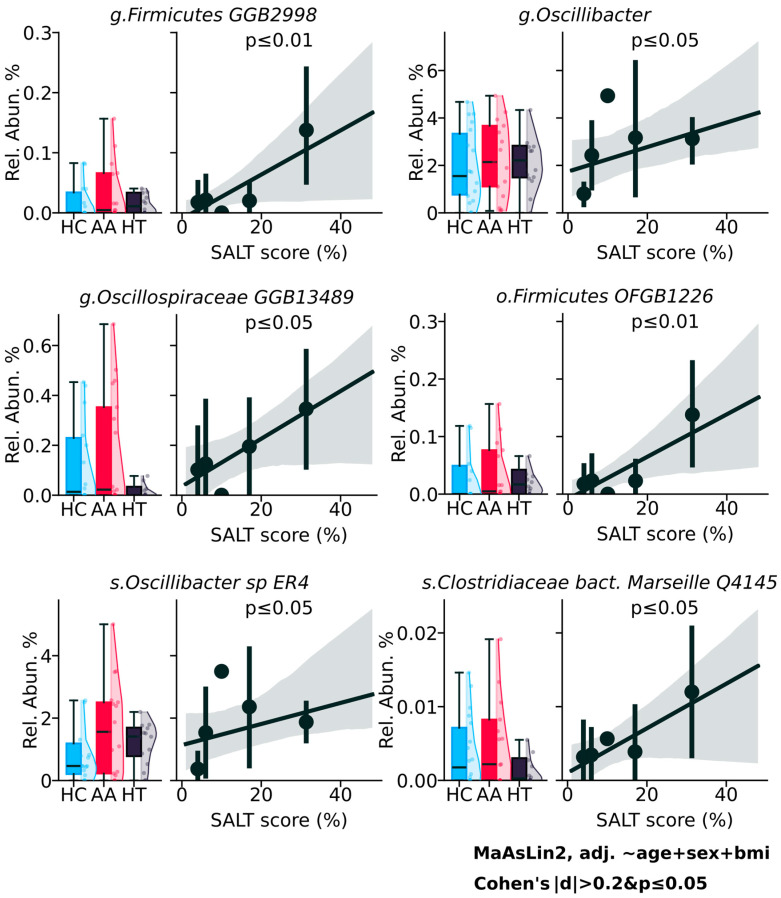
Gut microbial taxa associated with disease group and severity in alopecia areata.

Analysis of microbial abundance in relation to the Severity of Alopecia Tool (SALT) score revealed several taxa that exhibited a positive trend with AA severity (Figure 4), although the findings reflect statistical rather than clinical significance. At the genus level, *Firmicutes_GGB2998* (*p* ≤ 0.01), *Oscillibacter* (*p* ≤ 0.05), and *Oscillospiraceae_GGB13489* (*p* ≤ 0.05) showed increasing abundance with higher SALT scores, suggesting a possible link between these taxa and more extensive hair loss. At the order level, *Firmicutes_OFGB1226* (*p* ≤ 0.01), and at the species level, *Oscillibacter_sp_ER4* (*p* ≤ 0.05) and *Clostridiaceae_bacterium_Marseille_Q4145* (*p* ≤ 0.05) followed a similar pattern. While these correlations reached statistical significance, their biological or clinical relevance remains uncertain.

## 3. Discussion

This study reveals distinct microbial metabolic profiles associated with AA and HT, highlighting differences not in overall microbial diversity but in taxonomic composition and functional activity. Despite no significant differences in alpha and beta diversity metrics between groups, functional and taxonomic analyses revealed disease-specific alterations that may underlie divergent immune-metabolic interactions.

### 3.1. Known and Novel Gut Microbial Contributors to HT: Bacteroides fragilis and Microbacterium

Bacterial components such as lipopolysaccharide (LPS), a major constituent of gram-negative bacteria, may link gut microbiota to systemic inflammation. LPS can activate inflammatory signaling via JAK–STAT and AMPK-cPLA2 pathways, particularly under high-fat dietary conditions, promoting immune activation and barrier dysfunction [16]. The reduced butanoate metabolism observed in HT participants aligns with reports that excess iodine intake can reduce butyrate-producing bacteria. This reduction may disturb Th17/Treg balance, and contribute to autoimmune thyroid pathology [3]. Furthermore, specific microbial species such as *Bacteroides fragilis* have shown the ability to reverse Th1/Th2 imbalance through polysaccharide A (PSA)-dependent immune modulation. This supports the concept that microbial metabolites influence immune equilibrium [17]. Notably, *B. fragilis*, which was selectively enriched in HT, has been associated in previous studies with the activation of inflammatory pathways, including stimulation of the NLRP3 inflammasome and increased expression of cytokines such as IL-1β and IL-18 [4]. While these findings suggest a possible pro-inflammatory role, the exact contribution of *B. fragilis* to autoimmune processes remains incompletely understood. One proposed mechanism involves PSA, a surface capsule molecule. In experimental models, PSA has shown to influence immune responses by promoting IL-10—producing regulatory T cells and modulating host immune development [18]. Molecular mimicry by microbial antigens is thought to trigger autoimmunity by presenting epitopes structurally similar to host proteins [19]. In particular, *B. fragilis* has been shown to express proteins that mimic human ubiquitin, potentially leading to cross-reactive immune responses and loss of self-tolerance [20]. This dual role of immunoregulatory through PSA and immunostimulatory via molecular mimicry highlights the complex and context-dependent influence of *B. fragilis* in autoimmune conditions such as HT. Diet-related microbial shifts are also relevant. Western-style diets rich in fat and sugar have been associated with increased Firmicutes abundance and reduced microbial diversity. In contrast, high-polysaccharide diets are linked to more diverse communities and supportive of short-chain fatty acid (SCFA) production [21]. Lastly, the broader metabolic capacity of the microbiota surpasses that of the human host. Through fermentation of resistant starches, microbial communities generate diverse metabolites, including butyrate, with anti-inflammatory and immunomodulatory properties [22].

Among the taxa identified in our analysis, the genus *Microbacterium*, which has not previously been associated with thyroid diseases, showed increased abundance in individuals from HT group, with statistically significant differences compared to both HC (*p* ≤ 0.0001) and AA groups (*p* ≤ 0.001). This observation raises the hypothesis that *Microbacterium* could be involved in mechanisms such as molecular mimicry or chronic immune activation, which have previously been implicated in autoimmune thyroid disorders. Although the functional relevance of this genus in thyroid autoimmunity remains unclear, its consistent presence in HT samples suggests it may represent a microbial correlate worth further investigation.

These microbial and dietary factors may intersect with the disease-specific metabolic profiles observed in this study. For example, the reduction in butyrate and related fermentation pathways in HT could reflect impaired SCFA-mediated immune regulation, whereas the elevated biosynthetic and fermentative activity in AA may indicate a compensatory microbial response to acute inflammation. Together, these findings underscore the importance of microbial metabolism, particularly SCFAs, biotin, and LPS as potential contributors to the divergent immune landscapes of AA and HT.

### 3.2. Elevated Microbial Metabolic Activity and Functional Shifts in Alopecia Areata

In AA, the gut microbiome displayed a broadly elevated metabolic profile characterized by increased degradation of aromatic compounds, enhanced fermentation, and elevated amino acid biosynthesis. Notably, the abundance of protocatechuate degradation II and 4-hydroxyphenylacetate degradation pathways suggests intensified microbial processing of phenolic substrates, feeding into the β-ketoadipate pathway, a key route that channels aromatic intermediates into central metabolism via succinyl-CoA and acetyl-CoA production [23,24]. Furthermore, succinate fermentation to butanoate and assimilatory sulfate reduction IV were markedly elevated in AA. These pathways support microbial energy generation and biosynthetic sulfur assimilation, respectively, and have been previously linked to SCFA production and redox balance [25,26]. Of particular relevance, SCFAs, particularly butyrate, are key microbial metabolites produced through the fermentation of dietary polysaccharides by genera such as *Bacteroides*, *Bifidobacterium*, and *Lactobacillus*. SCFAs are known to enhance intestinal epithelial barrier function, reduce permeability, and exert downstream effects on peripheral sites, including modulation of dermal immunity and commensal composition [27]. A low-fiber diet and reduced abundance of SCFA-producing taxa can impair SCFA biosynthesis, potentially contributing to epithelial and immune disturbances relevant to AA [28]. The observed upregulation of L-methionine biosynthesis II and L-cysteine biosynthesis VI further supports the shift toward sulfur-containing amino acid metabolism, potentially contributing to methylation pathways [29,30]. Importantly, taxa such as *Enterobacter*, *Blautia glucerasea*, and *Mediterraneibacter* were significantly correlated with these functional shifts. Specifically, both *Enterobacter* and *Mediterraneibacter* showed strong positive correlations with TCA cycle VI (Helicobacter-type) (r = 0.5), indicating their active involvement in central energy metabolism under microaerophilic conditions. *Blautia glucerasea* was also positively linked with protocatechuate degradation II, sulfur amino acid biosynthesis, and assimilatory sulfate reduction, supporting its multifunctional role in aromatic degradation and biosynthetic flux, suggesting taxon-specific contributions to the observed metabolic enhancement. In contrast, HT was associated with a more constrained metabolic signature. Central energy-producing pathways, including TCA cycle VII (acetate producers), were downregulated, while UDP-N-acetylglucosamine-derived O-antigen biosynthesis was upregulated, suggesting increased microbial investment in surface carbohydrate structures [31].

### 3.3. Potential but Inconclusive Microbial Correlates of AA Severity

Previous research has shown that high SALT scores, reflecting greater severity of AA, are associated with notable changes in gut microbial beta diversity, including enrichment of *Alistipes*, *Bacteroides*, and *Barnesiella*, and a concurrent reduction in beneficial taxa such as *Lachnospiraceae* and *Ruminococcaceae*. These shifts were accompanied by an increase in circulating CCR6^+^CD4^+^ T cells, suggesting possible immunological consequences linked to the altered microbial composition [32]. Several microbial taxa, illustrated in Figure 4, demonstrated a positive correlation with AA severity as measured by the SALT score. While these associations reached statistical significance, their clinical applicability remain unclear. These findings suggest microbial patterns that may be related to disease severity, but their clinical significance remains uncertain and requires further validation and functional studies to determine their relevance in the context of alopecia areata pathogenesis.

This study has several important limitations that should be acknowledged. First, our analysis was unable to detect polysaccharide biosynthesis pathways, including those involved in PSA production by *B. fragilis*. The HUMAnN tool, which relies on the MetaCyc database, does not annotate capsule formation or complex polysaccharide gene clusters. As a result, while PSA-mediated immune modulation remains a theoretically plausible mechanism, our analysis could not confirm its presence or functional relevance. Further molecular-level studies are needed to determine which of these mechanisms, if any, are active in thyroid autoimmunity. Second, the consistent enrichment of *Microbacterium* sp. *T32* in HT and AA samples requires cautious interpretation. *Microbacterium* is not a typical gut commensal and may represent an environmental taxon originating from water or soil, or even a reagent contaminant [33]. Further validation in independent cohorts, supported by qPCR confirmation and appropriate decontamination controls, is essential before any mechanistic role can be inferred. Third, the modest cohort size limits statistical power and increases the risk of type I error, especially under multiple testing. Finally, several HT patients were receiving levothyroxine, which may influence metabolism and gut microbiota. This potential effect should be considered when interpreting the findings.

## 4. Materials and Methods

### 4.1. Study Subjects and Demographics

The demographic characteristics of the study cohort are described in Table 1. A total of 51 subjects aged 18–65 years were divided into three groups: patients diagnosed with alopecia areata (*n* = 17), patients diagnosed solely with Hashimoto’s thyroiditis (*n* = 16), and healthy controls (*n* = 18). Patients were screened for thyroid disease using a binary (yes/no) assessment based on their medical records. Exclusion criteria included the use of antibiotics or probiotics within the last six months.

All patients underwent biochemical testing. The panel included C-reactive protein (CRP), antinuclear antibodies (ANA), anti-thyroglobulin (anti-TG), anti-thyroid peroxidase (anti-TPO) antibodies, free thyroxine (FT4), free triiodothyronine (FT3), and tissue transglutaminase (TTG) antibodies. These tests were used to exclude other autoimmune or inflammatory diseases and confirm the diagnosis of autoimmune thyroiditis consistent with Hashimoto thyroiditis. Some patients with Hashimoto’s thyroiditis were receiving thyroid hormone replacement therapy. All patients who were prescribed hormone replacement therapy by their physicians were treated with levothyroxine, at doses ranging from 25 to 100 µg per day. Fecal samples were collected after obtaining informed consent from all participants. The study was conducted under the approval of the Ethics Committee of the Center for Life Sciences, National Laboratory Astana (#05-2022) and in accordance with the Declaration of Helsinki.

### 4.2. Sample Collection and Processing

Fecal samples were collected into DNA/RNA Shield Fecal Collection tubes (#R1101, Zymo Research, Tustin, CA, USA) and stored at +4 °C until processing. Total genomic DNA was isolated from fecal samples using the ZymoBIOMICS DNA Microprep Kit (#4300, Zymo Research, USA). Extracted DNA quality was assessed using Nanodrop and 1.0% agarose gel electrophoresis. Sequencing was performed using the Illumina 6000 platform following the manufacturer’s standard protocols (Novogene Laboratory, Beijing, China). Raw sequencing reads were processed using KneadData v0.12.0 for quality control and removal of host DNA contamination. Taxonomic profiling was performed using MetaPhlAn with the mpa_vJun23_CHOCOPhlAnSGB_202403 database. Functional pathway profiling was conducted using HUMAnN v3.8, with gene family abundances mapped to the UniRef90 protein database for downstream analysis. All analyses were carried out using default parameters unless otherwise specified.

### 4.3. Statistical Analysis

Statistical analysis and visualization were performed in Python v3.12 using NumPy v2.0.1, SciPy v1.15.1, statsmodels v0.14.4, Matplotlib v3.10.0, and seaborn v0.13.2 packages. Biodiversity analysis was performed using scikit-bio v0.6.3. The baseline characteristics of the groups were compared using the generalized linear model (GLM), adjusting for age, sex and BMI. Differential analysis was performed using MaAsLin2 adjusting for age, sex and BMI. For the differential analysis, markers were considered significant at *p* < 0.05 (q < 0.25, FDR) and Cohen’s d effect size |d| > 0.2. Only features with a prevalence of at least 50% (in any group) were considered for analysis. Between-group diversity was assessed using Bray–Curtis and Jaccard distances. The significance of grouping was assessed using Adonis2 function from the vegan v2.7.1 package in R v4.4.2. Principal coordinate analysis (PCoA) was used to visualize compositional differences between samples. Within-sample diversity was assessed using the Chao1 and Shannon indices at species-level genome bins (SGBs). Correlation analysis between marker taxa, pathways, and clinical parameters was performed using Spearman’s coefficient.

## 5. Conclusions

In conclusion, *Bacteroides fragilis* was selectively enriched in Hashimoto’s thyroiditis, suggesting a key role in immune modulation through mechanisms like PSA production and molecular mimicry. Its dual immunoregulatory and pro-inflammatory potential has been proposed in previous studies, suggesting possible relevance in thyroid autoimmunity and warranting further investigation. The elevated metabolic activity observed in alopecia areata, including increased fermentation and biosynthesis, may reflect an active microbial response to acute immune stimulation, consistent with the episodic and immune-driven nature of the disease. In contrast, the reduced metabolic output and enhanced immunogenic carbohydrate biosynthesis seen in Hashimoto’s thyroiditis align with its gradual onset and chronic inflammatory progression. These distinct microbial patterns may not only reflect the biology of each disease but also offer potential targets for future therapies aimed at restoring microbial balance. Targeting specific microbial pathways or taxa, such as SCFA restoration or suppression of inflammatory species like *B. fragilis*, may offer new therapeutic opportunities for both conditions.

## Figures and Tables

**Table 1:** Subject’s demographic characteristics.

Characteristics	Alopecia Areata	Hashimoto Thyroiditis	Healthy Control
*n*	17	16	18
Age, years, SD	38.47 ± 5.14	37.06 ± 9.15	28.56 ± 5.14
Sex (Female/Male)	10/7	14/2	12/6
BMI	25.43 ± 4.83	24.41 ± 3.18	22.74 ± 4.53
Disease duration, years, SD	8 ± 6.13	8.3 ± 4.44	-
An elevated anti-TPO antibody level (>35 IU/mL)	4 (23%)	14 (87.5%)	-
Thyroid hormone replacement therapy	3 (18%)	9 (56%)	-
Family history			
Alopecia areata	5 (29%)	2 (12.5%)	-
Hashimoto thyroiditis	2 (12%)	7 (44%)	-

## Data Availability

Raw sequencing data are available at the National Center for Biotechnology Information under the accession number PRJNA1299156.

## Supplementary Materials

The following supporting information can be downloaded at: https://www.mdpi.com/article/10.3390/ijms26178724/s1.

## Abbreviations

The following abbreviations are used in this manuscript:

AA	Alopecia areata
HT	Hashimoto’s thyroiditis
HC	Healthy control
IL-1β, IL-18, IL-10	Interleukin 1 beta, 18, 10
JAK-STAT	Janus kinase-signal transducer and activation of transcription
LPS	Lipopolysaccharide
PSA	Polysaccharide A
SALT	Severity of Alopecia areata
SCFA	Short-chain fatty acids
TCA	Tricarboxylic Acid
Th1, Th2, Th17, Treg	T helper cell types 1, 2, 17, Regulatory T cells

## References

[1] Caturegli P., De Remigis A., Rose N.R. (2014). Hashimoto thyroiditis: Clinical and diagnostic criteria. Autoimmun. Rev..

[2] Fröhlich E., Wahl R. (2017). Thyroid Autoimmunity: Role of Anti-thyroid Antibodies in Thyroid and Extra-Thyroidal Diseases. Front. Immunol..

[3] Gong B., Meng F., Wang X., Han Y., Yang W., Wang C., Shan Z. (2024). Effects of iodine intake on gut microbiota and gut metabolites in Hashimoto thyroiditis-diseased humans and mice. Commun. Biol..

[4] Gong B., Wang C., Meng F., Wang H., Song B., Yang Y., Shan Z. (2021). Association Between Gut Microbiota and Autoimmune Thyroid Disease: A Systematic Review and Meta-Analysis. Front. Endocrinol..

[5] Pratt C.H., King L.E., Messenger A.G., Christiano A.M., Sundberg J.P. (2017). Alopecia areata. Nat. Rev. Dis. Primers.

[6] Shi Q., Duvic M., Osei J.S., Hordinsky M.K., Norris D.A., Price V.H., Amos C.I., Christiano A.M., Mendoza T.R. (2013). Health-Related Quality of Life (HRQoL) in alopecia areata patients-a secondary analysis of the National Alopecia Areata Registry Data. J. Investig. Dermatol. Symp. Proc..

[7] Chu S.Y., Chen Y.J., Tseng W.C., Lin M.W., Chen T.J., Hwang C.Y., Chen C.C., Lee D.D., Chang Y.T., Wang W.J. (2011). Comorbidity profiles among patients with alopecia areata: The importance of onset age, a nationwide population-based study. J. Am. Acad. Dermatol..

[8] Belkaid Y., Hand T.W. (2014). Role of the microbiota in immunity and inflammation. Cell.

[9] Honda K., Littman D.R. (2016). The microbiota in adaptive immune homeostasis and disease. Nature.

[10] Rooks M.G., Garrett W.S. (2016). Gut microbiota, metabolites and host immunity. Nat. Rev. Immunol..

[11] Wu H.J., Ivanov I.I., Darce J., Hattori K., Shima T., Umesaki Y., Littman D.R., Benoist C., Mathis D. (2010). Gut-residing segmented filamentous bacteria drive autoimmune arthritis via T helper 17 cells. Immunity.

[12] Ivanov I.I., Atarashi K., Manel N., Brodie E.L., Shima T., Karaoz U., Wei D., Goldfarb K.C., Santee C.A., Lynch S.V. (2009). Induction of intestinal Th17 cells by segmented filamentous bacteria. Cell.

[13] Gao L., Li W., Song Q., Gao H., Chen M. (2024). The genetic link between thyroid dysfunction and alopecia areata: A bidirectional two-sample Mendelian randomization study. Front. Endocrinol..

[14] Simakou T., Butcher J.P., Reid S., Henriquez F.L. (2019). Alopecia areata: A multifactorial autoimmune condition. J. Autoimmun..

[15] Li D., Liang G., Calderone R., Bellanti J.A. (2019). Vitiligo and Hashimoto’s thyroiditis: Autoimmune diseases linked by clinical presentation, biochemical commonality, and autoimmune/oxidative stress-mediated toxicity pathogenesis. Med. Hypotheses.

[16] Chang C.C., Sia K.C., Chang J.F., Lin C.M., Yang C.M., Huang K.Y., Lin W.N. (2019). Lipopolysaccharide promoted proliferation and adipogenesis of preadipocytes through JAK/STAT and AMPK-regulated cPLA2 expression. Int. J. Med. Sci..

[17] Mazmanian S.K., Liu C.H., Tzianabos A.O., Kasper D.L. (2005). An immunomodulatory molecule of symbiotic bacteria directs maturation of the host immune system. Cell.

[18] Erturk-Hasdemir D., Kasper D.L. (2018). Finding a needle in a haystack: *Bacteroides fragilis* polysaccharide A as the archetypical symbiosis factor. Ann. N. Y. Acad. Sci..

[19] English J., Patrick S., Stewart L.D. (2023). The potential role of molecular mimicry by the anaerobic microbiota in the aetiology of autoimmune disease. Anaerobe.

[20] Stewart L., Edgar J.D.M., Blakely G., Patrick S. (2018). Antigenic mimicry of ubiquitin by the gut bacterium *Bacteroides fragilis*: A potential link with autoimmune disease. Clin. Exp. Immunol..

[21] De Filippo C., Cavalieri D., Di Paola M., Ramazzotti M., Poullet J.B., Massart S., Collini S., Pieraccini G., Lionetti P. (2010). Impact of diet in shaping gut microbiota revealed by a comparative study in children from Europe and rural Africa. Proc. Natl. Acad. Sci. USA.

[22] Fröhlich E., Wahl R. (2019). Microbiota and Thyroid Interaction in Health and Disease. Trends Endocrinol. Metab..

[23] Harwood C.S., Parales R.E. (1996). The beta-ketoadipate pathway and the biology of self-identity. Annu. Rev. Microbiol..

[24] Díaz E., Ferrández A., Prieto M.A., García J.L. (2001). Biodegradation of aromatic compounds by Escherichia coli. Microbiol. Mol. Biol. Rev..

[25] Kenealy W.R., Waselefsky D.M. (1985). Studies on the substrate range of Clostridium kluyveri; the use of propanol and succinate. Arch. Microbiol..

[26] Berndt C., Lillig C.H., Wollenberg M., Bill E., Mansilla M.C., de Mendoza D., Seidler A., Schwenn J.D. (2004). Characterization and reconstitution of a 4Fe-4S adenylyl sulfate/phosphoadenylyl sulfate reductase from Bacillus subtilis. J. Biol. Chem..

[27] Martin-Gallausiaux C., Marinelli L., Blottière H.M., Larraufie P., Lapaque N. (2021). SCFA: Mechanisms and functional importance in the gut. Proc. Nutr. Soc..

[28] Liu Z., Liu X. (2023). Gut microbiome, metabolome and alopecia areata. Front. Microbiol..

[29] Soda K. (1987). Microbial sulfur amino acids: An overview. Methods Enzymol..

[30] Doherty N.C., Shen F., Halliday N.M., Barrett D.A., Hardie K.R., Winzer K., Atherton J.C. (2010). In Helicobacter pylori, LuxS is a key enzyme in cysteine provision through a reverse transsulfuration pathway. J. Bacteriol..

[31] Samuel G., Reeves P. (2003). Biosynthesis of O-antigens: Genes and pathways involved in nucleotide sugar precursor synthesis and O-antigen assembly. Carbohydr. Res..

[32] Bain K.A., Nichols B., Moffat F., Kerbiriou C., Ijaz U.Z., Gerasimidis K., McInnes I.B., Åstrand A., Holmes S., Milling S.W.F. (2022). Stratification of alopecia areata reveals involvement of CD4 T cell populations and altered faecal microbiota. Clin. Exp. Immunol..

[33] Salter S.J., Cox M.J., Turek E.M., Calus S.T., Cookson W.O., Moffatt M.F., Turner P., Parkhill J., Loman N.J., Walker A.W. (2014). Reagent and laboratory contamination can critically impact sequence-based microbiome analyses. BMC Biol..

