# Supplementary figures and images for: Bacteroides fragilis and Microbacterium as Microbial Signatures in Hashimoto’s Thyroiditis
Source: Int J Mol Sci. 2025 Sep 7;26(17):8724. doi: 10.3390/ijms26178724 (PMC12429219; doi:10.3390/ijms26178724)

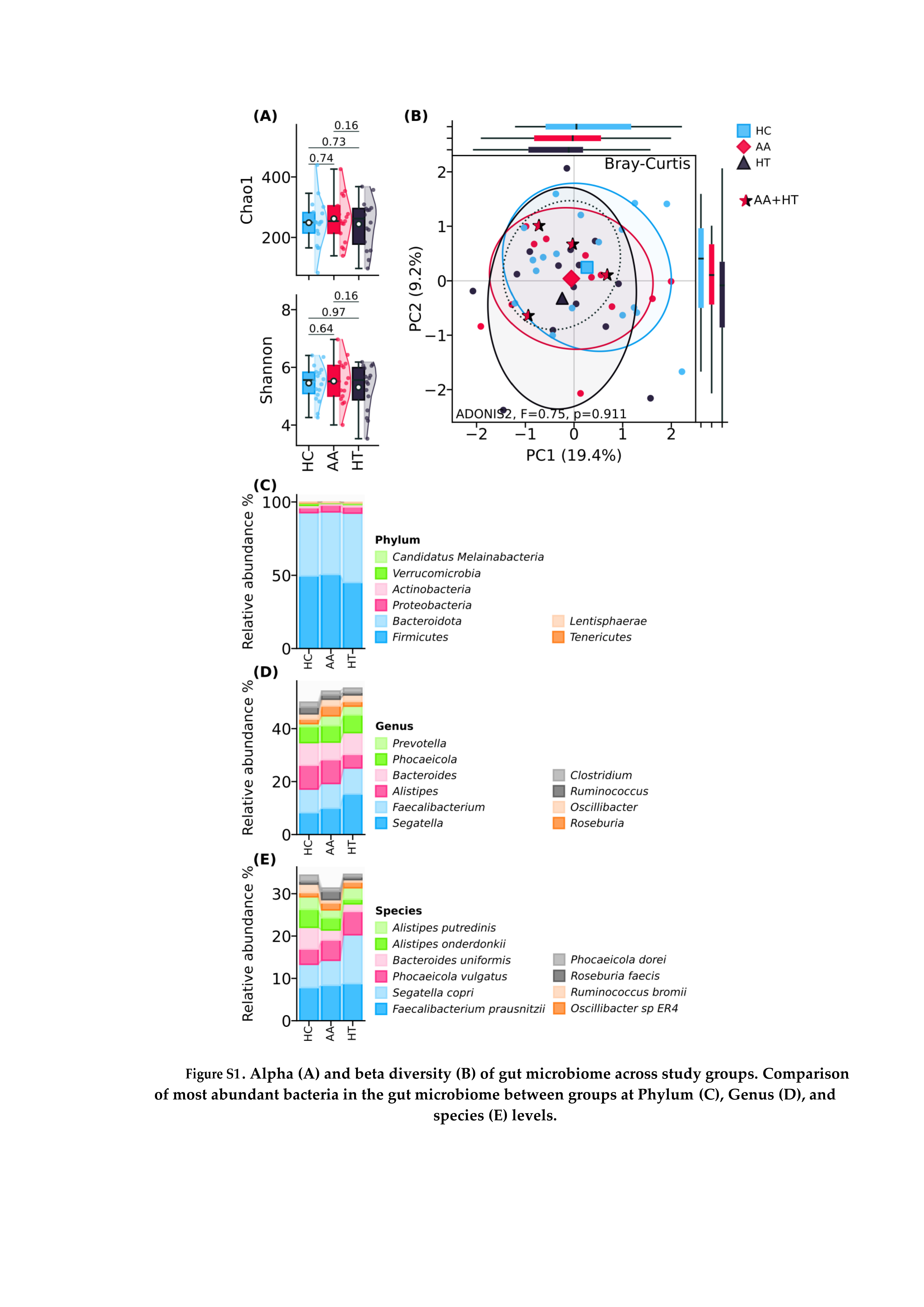

Supplement: Supplementary file 1 [file ijms-26-08724-s001.zip › Figure S1.tif]
